# Microarray-Based Maps of Copy-Number Variant Regions in European and Sub-Saharan Populations

**DOI:** 10.1371/journal.pone.0015246

**Published:** 2010-12-16

**Authors:** Christian Vogler, Leo Gschwind, Benno Röthlisberger, Andreas Huber, Isabel Filges, Peter Miny, Bianca Auschra, Attila Stetak, Philippe Demougin, Vanja Vukojevic, Iris-Tatjana Kolassa, Thomas Elbert, Dominique J.-F. de Quervain, Andreas Papassotiropoulos

**Affiliations:** 1 Department of Psychology, University of Basel, Basel, Switzerland; 2 Department Biozentrum, University of Basel, Basel, Switzerland; 3 Department of Medicine, University of Basel, Basel, Switzerland; 4 Center of Laboratory Medicine, Cantonal Hospital, Aarau, Switzerland; 5 Department of Biomedicine, University Children's Hospital, Basel, Switzerland; 6 Department of Psychology, University of Konstanz, Konstanz, Germany; Aarhus University, Denmark

## Abstract

The genetic basis of phenotypic variation can be partially explained by the presence of copy-number variations (CNVs). Currently available methods for CNV assessment include high-density single-nucleotide polymorphism (SNP) microarrays that have become an indispensable tool in genome-wide association studies (GWAS). However, insufficient concordance rates between different CNV assessment methods call for cautious interpretation of results from CNV-based genetic association studies. Here we provide a cross-population, microarray-based map of copy-number variant regions (CNVRs) to enable reliable interpretation of CNV association findings. We used the Affymetrix Genome-Wide Human SNP Array 6.0 to scan the genomes of 1167 individuals from two ethnically distinct populations (Europe, N = 717; Rwanda, N = 450). Three different CNV-finding algorithms were tested and compared for sensitivity, specificity, and feasibility. Two algorithms were subsequently used to construct CNVR maps, which were also validated by processing subsamples with additional microarray platforms (Illumina 1M-Duo BeadChip, Nimblegen 385K aCGH array) and by comparing our data with publicly available information. Both algorithms detected a total of 42669 CNVs, 74% of which clustered in 385 CNVRs of a cross-population map. These CNVRs overlap with 862 annotated genes and account for approximately 3.3% of the haploid human genome.

We created comprehensive cross-populational CNVR-maps. They represent an extendable framework that can leverage the detection of common CNVs and additionally assist in interpreting CNV-based association studies.

## Introduction

Copy Number Variations (CNVs) have been recently receiving growing attention as a steadily increasing number of CNVs in the human genome has been identified [1], [2] and successfully linked to a variety of medical conditions [3], [4], [5], [6]. CNVs can cover whole gene loci and may have dramatic impact on protein expression levels through altering gene dosage or disrupting coding sequences [7], [8]. Hence it is crucial to investigate copy number states as a source of genetic variation in genome-wide association studies (GWAS). In addition, accounting for the presence of CNVs could also improve SNP genotyping results. Rejection of SNPs in GWAS due to violation of Hardy-Weinberg-Equilibrium might for example stem from individuals that are hemizygous for a specific allele and are wrongly assigned the homozygous genotype call.

Although increasing efforts have been put into the refinement of methods for the accurate and reliable detection of CNVs [9], [10], [11], [12], available methods suitable for high-density oligonucleotide SNP arrays still lack sufficient sensitivity and specificity [13]. In order to improve detection methods for Copy Number Events, standard maps providing information on genomic regions that are prone to structural variation are needed. Such maps containing information about hotspots for CNV-formation can provide prior knowledge in Bayesian terms about genomic localization and frequency of occurrence of CNVs. Incorporation of these priors leads to a considerably reduced marker set that either facilitates faster detection of common CNVs or allows for a more precise CNV-analysis.

Like microsatellites and single-nucleotide polymorphisms (SNPs), CNVs represent a specific form of genetic variation. Global distribution of CNVs largely accords with population structure analysis for SNP data sets [14]. Findings from population genetics show that global human genetic variation is greatly influenced by geographical migration [15], [16] and that genetic diversity within populations decreases with increasing geographic distance from Africa [17]. Hence, differences in genetic diversity between populations should not be neglected in CNV analysis.

In this study we provide a cross-population, microarray-based map of copy-number variant regions (CNVRs) to enable reliable interpretation of CNV association findings.

**Figure 1:**
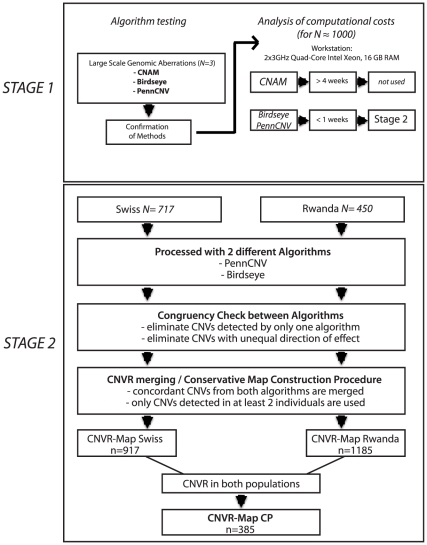
Workflow chart of the map construction procedure.

To account for differences in variation patterns between populations, we created two population-specific CNVR-maps and a cross-populational standard CNVR-map (Figure 1). In order to decrease the number of false positive findings, we built CNVR-maps using only convergent CNVs detected by two different algorithms found in at least two individuals. On the other hand we investigated hundreds of individuals, thus boosting detection sensitivity. To corroborate our findings, we also processed two subsamples with the Illumina 1M-Duo BeadChip and the Nimblegen 385K aCGH array. Further we checked our CNVR maps against previously published data.

The CNVR-maps represent an extendable framework that can leverage the detection of common CNVs and additionally assist in interpreting CNV-based association studies.

## Results

Our goal was to create CNVR maps that can provide references for population specific frequencies and the genetic localization of common CNVs as assessed by commonly used SNP arrays. Therefore we analyzed a Sub-Saharan African population (N = 450) and a sample of European ancestry (N = 717). We applied two different copy number finding algorithms (Birdseye and PennCNV) to data generated by the Affymetrix Genome-Wide Human SNP Array 6.0, an array that was specifically designed to assess copy number states throughout the genome [18].

### Gross Validation

**Figure 2:**
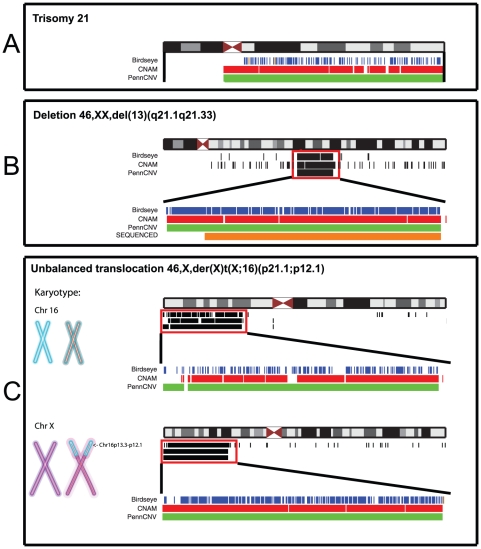
Detection of large-scale genomic variants with CNAM, PennCNV and Birdseye and the Affymetrix SNP Array 6.0. Schematic representation of the three samples with large-scale genomic aberrations used for gross validation of methods. Results of the different CNV-Finding algorithms are shown on a chromosome wide level, black and colored bars indicate CNVs. For B and C a more detailed view of the large-scale aberration regions (red boxes) is given. Orange bars for the Birdseye algorithm in A and C represent CNVs with opposite direction of effect (gain vs. loss). A. Trisomy 21. (Due to repetitive and no known coding sequences on the p-arm of chromosome 21, the Affymetrix GeneChip 6.0 lacks markers in this genomic region. Therefore changes in copy number in this region are not detectable.) B. Well defined familial 14.5 Mb deletion 46,XX,del(13)(q21.1q21.33) with sequenced breakpoints (orange bar) [32]. C. Partial terminal duplication of the short arm of chromosome 16 and partial terminal deletion of the short arm of the X-chromosome. Conventional karyotyping confirmed an unbalanced translocation 46,X,der(X)t(X;16)(p21.1;p12.1).

For evaluation of CNV-finding algorithms we checked whether CNAM, PennCNV and Birdseye [9], [10], [19] would reliably detect large-scale genomic aberrations. Therefore we processed three DNA samples with verified large-scale structural variations using the Affymetrix SNP Chip 6.0. Generally, all three algorithms were able to detect the structural variations yielding comparable results. A striking difference between algorithms is the degree of fragmentation of detected CNVs. Especially, PennCNV and Birdseye, the two algorithms subsequently chosen to process the data of the two populations, differ significantly (Figures 1, 2).

### Stage 2 Analysis

We decided not to use CNAM for further analysis because this algorithm is computationally expensive and dependent on subjective cut-off values, which have to be defined by the user after visual inspection of data. Processing a sample with the CNAM algorithm, which is of equal size to the one used in this study, would take up to 4 weeks (2x3GHz Quad-Core Intel Xeon, 16 GB RAM), whereas applying PennCNV and Birdseye to this amount of data is accomplishable in a few days.

**Figure 3:**
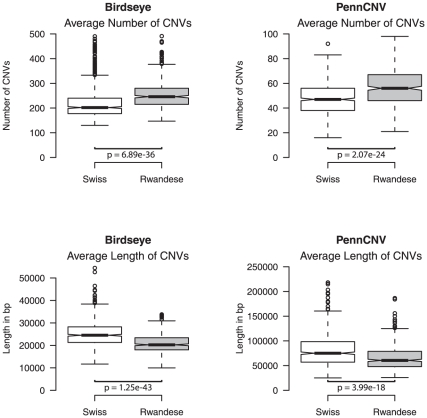
Differences in average length and number of CNVs between populations for Birdseye and PennCNV. Upper left: Average length of CNVs Birdseye Upper right: Average length of CNVs PennCNV Lower left: Average Number of CNVs found per individual Birdseye Lower right: Average Number of CNVs found per individual PennCNV Boxes represent 25–75% of data points, whiskers indicate 1.5 x the interquartile range, outliers are depicted by the small circles.

Therefore we processed the two populations with the computationally more efficient algorithms PennCNV and Birdseye. Interestingly, albeit PennCNV and Birdseye generally report an unequal amount of copy number events, population differences in CNV characteristics are well reflected by the data. Both algorithms consistently report a higher number of CNVs that are of shorter length in the African sample (Figure 3). This is in accordance with findings that haplotype blocks in Africans are significantly smaller and show higher degrees of diversity than in non-African samples [20]. The finding of higher frequencies of CNVs in the Rwandese sample is conform with the expected generally higher genetic diversity in Sub-Saharan African populations as also reflected by the difference in genome-wide heterozygosity in SNPs (mean_Swiss  = 26.24, mean_Rwanda = 28.01, W = 7518, p = 2.2e–157).

### Construction of the CNVR-Map

**Figure 4:**
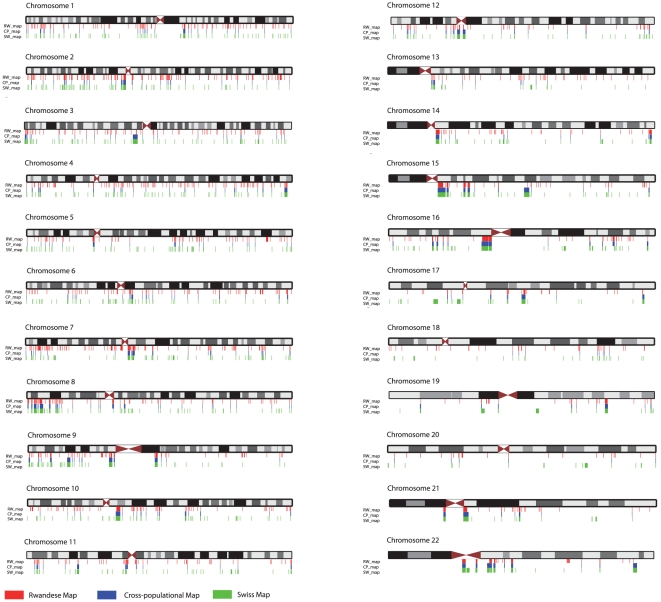
Whole genome view for the three CNVR maps. Depicted are schematic representations and banding patterns of autosomal chromosomes in single-chromatid formation. The colored tick-marks represent CNVRs (red  =  Rwandese map, blue  =  Cross-populational map, green  =  Swiss map). Figure was generated using the UCSC genome browser.

Albeit obvious differences in sensitivity and specificity between Birdseye and PennCNV, it is possible to use the combination of both algorithms to identify Copy Number Variable Regions (CNVRs). For this purpose we first scanned data on an individual basis for CNVs. The total number of CNVs reported by the Birdseye algorithm for both samples was 310835 and 60532 for the PennCNV algorithm, respectively. We analyzed which CNVs were reported by both algorithms and overlapped, merging these into one CNV. Also direction of effect (gain vs. loss) had to be equal for CNVs to be used for CNVR creation. This procedure was applied to the Swiss and the Rwandese sample and yielded a total of 42669 CNVs. A more detailed description of this process can be found in the Figure S1. The cross-populational CNVR map (CP-map, Figure 4) was constructed using only CNVRs that were common to both the Rwandese and the Swiss sample. This CP-map comprises a total of 385 common CNVRs on 22 autosomes (sex chromosomes were excluded from analysis). The Swiss and the Rwandese CNVR map contain a total of 917 and 1185 CNVRs, respectively. For a detailed description of the map creation procedures see Methods S1.

**Figure 5:**
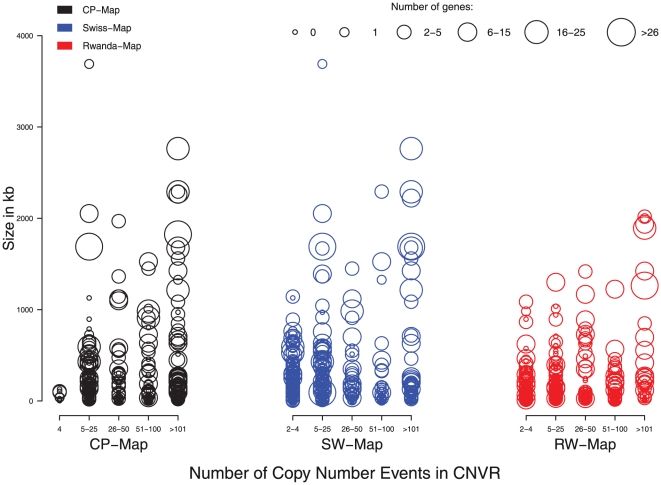
CNVR-length, gene content and frequency distributions. Plot is depicting CNVR-length, gene content and frequency distributions. CNVRs are plotted according to CNVR-map (color), length (y-axis), frequency of CNVs per CNVR (x-axis, at least 2 overlapping CNVs had to be present to form a CNVR in the population specific maps, a CNVR in the CP-Map is constructed of at least 2×2 CNV events) and number of RefSeq genes affected (circle size).

Maps were screened for genes that overlap with CNVRs. A total of 862 genes were affected by CNVRs in the CP-Map (e.g. *MAPK1* on chromosome 22: 20443947-20551970, *NEGR1* on chromosome 1: 71641213-72520993, *PARK2* on chromosome 6: 161688580-163068824). Complete lists of genes affected by CNVRs in all three maps are given in Table S1. A general overview of CNVR frequencies and number of genes located within the CNVRs is given in Figure 5.

We also analyzed the percentages of genes that were detected in both the Sw-map and the Rw-map or that were specific for either population. A total of 1585 genes have been found to overlap with CNVRs. Approximately one third (34%) of these genes are found to map to CNVRs in both populations. One fourth (25%) were found to be affected by copy number variation in the Rwandese sample only, whereas 41% of these genes were found to be copy number variable only in the Swiss population. A detailed overview and lists of genes are available in Table S1.

Comparison with data downloaded from the Database of Genomic Variants (DGV – current version: hg18.v7.aug.2009) revealed substantial overlap between our CP-map and entries of single CNVs in the DGV. Out of 56497 autosomal copy number events reported in the DGV, 11545 clustered in CNVRs as defined by the CP-map. Separate comparisons for the CNVR maps created on the single population basis (SW-map and RW-map) resulted in 13773 overlapping CNVs for the Rwandese based CNVR-map and 14369 for the Swiss based CNVR-map (Table S1). We restricted our analysis to CNVRs, choosing a conservative approach, which does not take rare events into account. Since CNVs reported in the DGV are measured by a variety of methods such as high-density aCGH and direct sequencing in different populations this finding indicates that a substantial part of copy number variation is detectable by high density SNP-Chips. Our CP-map contains only 3 previously undetected CNVR regions that do not contain any known genes. (chr2:57638382-57684603, chr2:221793857-221798933 and chr20:16610218-16618759).

The SW-map and the RW-map contain 80 and 191 CNVRs, respectively, that have not yet been reported to the DGV. We further compared CN-events that were detected by both algorithms to a map comprising 229 autosomal CNVRs constructed through analyzing 700 Chinese individuals with the Affymetrix 500K processed with the CNAT algorithm [21]. Comparison between CP-Map and the CNVRs detected in the Chinese sample yielded 98 overlapping regions. We found a total of 141 CNVRs that overlapped when comparing the Chinese map to the SW-map and 124 overlapping events when compared to the RW-map.

We also could observe overlaps between our CP-map and a list of 161 autosomal CNVRs identified with CNV-seq that used direct sequencing reads of Dr. C. Venter and Dr. J. Watson [22]. 79 out of the 161 CNVRs clustered in the CP-map. Separate comparisons revealed a total 84 overlapping CNVRs with the SW-map and 77 overlaps with the RW-map.

For further validation of our CP-map we processed 13 individuals with the Illumina Human 1M-Duo BeadChip, (7 Swiss, 6 RWS) and 9 samples of the Swiss population with the Nimblegen 385K aCGH array. We detected a total of 930 CNVs applying PennCNV on the Illumina 1M-Duo BeadChip. Comparison of these 930 CNVs to the CP-map showed 501 overlapping copy number events with the CNVRs in the map.

The 385K data from Swiss individuals was analyzed using the wuHMM algorithm [12]. Results of aCGH analysis for the nine investigated samples yielded a total of 264 copy number events of which 180 clustered in the SW-map and 162 in the CP-map. PennCNV reported a total of 346 CNVs for these nine samples. Interestingly, when we compared how many CNV events in these 9 individuals were detected by both methods in the same individual, only a total of 55 CNVs were detected by aCGH that overlapped with CNVs reported by PennCNV. This is another hint that current methods for CNV detection lack sensitivity and need further refinement. A detailed view of overlapping events can be found in Table S2.

As segmental duplications are known to play an important role in CNV formation [23], [24], [25], [26], we downloaded the *Segmental Dups* Track from UCSC table browser (Mar.2006 assembly, NCBI36/hg18) and analyzed whether the CNVRs as reported in our maps overlap with known segmental duplications. Out of 36119 annotated segmental duplications mapping to “non-random” chromosomal regions, 8911 overlapped with 178 CNVRs comprised in the CP-Map.

## Discussion

First, we note that all tested algorithms were able to detect large-scale genomic aberrations ranging from a 14 Mb deletion to a whole chromosome triplication. We therefore conclude that the SNP-Array under study can be used in cytogenetic research. Yet, as depicted in Figure 1, CNV-finding algorithms may vary considerably in sensitivity and specificity [13] and therefore we recommend a combination of different algorithms to facilitate interpretation of findings. Given this varying accuracy of different CNV-detection algorithms in large-scale genomic aberrations, cautiousness is indicated in interpreting findings from whole genome CNV screenings based on SNP-genotyping data. Common CNVs can comprise only few markers decreasing significantly the signal-to-noise ratio as compared to large-scale genomic aberrations. In order to improve reliability of CNV detection in single individuals, we suggest that detection algorithms should be capable of exploiting prior knowledge about CNV base rates in a given genomic region. To provide information on localization of CNVRs and probability measures of CNV occurrence contained therein, we set out to create standard maps of common CNVRs. Given that CNV-formation rate is considerably higher than the mutation rate for single base pairs [27], it is advisable to create population-specific priors to account for varying degrees of genetic diversity.

To decrease the probability that false positive CNV-calls are used for generating the CNVR-maps, we only took concordant CNV-calls of two independent algorithms into account that overlapped in at least two individuals. On the other hand we screened hundreds of individuals, thus increasing sensitivity.

The maps created this way contain information on CNV hotspots and frequency distributions of CNVs. They represent first drafts of population-specific maps as well as a cross-populational map, probably comprising phylogenetically older CNVRs. We report a substantial overlap with CNVR-regions created from previously published data and also were able to validate our maps using different array types, which vary in resolution and their mode of operation due to different chip-architectures.

We argue, that detection accuracy of CNVs can benefit from information on base rates of CNVs in the general population as provided in our CNVR-maps. Two ways to integrate prior knowledge about common CNVRs are plausible: On the one hand, the restriction of CNV-analysis to markers known to be located in CNVRs allows the use of computationally more expensive algorithms that could outperform current methods. On the other hand information gained from CNVR-maps can serve as prior probabilities in Bayesian terms that can be incorporated in CNV-detection algorithms. In order to alleviate assessing how common CNVs relate to variation in phenotypes, higher sensitivity and specificity rates in CNV-detection are needed. We suggest that the use of prior knowledge about CNVRs as provided by our maps, could yield the necessary refinement of methods. We note that our CNVR-maps do not depict structural variations on the p-arms of chromosome 13, 14, 15, 21 and 22 due to lack of markers in these areas on the Affymetrix 6.0 SNP Array. These regions only contain non-chromosome specific highly repetitive DNA sequences and are usually not covered by microarrays. It is important to stress that the array design for the Affymetrix Human SNP Array 6.0 relied mainly on variation detected in non-African populations an thus our Rw-map might underestimate the total variation present in the Rwandese sample. Specially designed custom arrays that take population specificity into account by making use of information provided by large scale sequencing endeavors such as the 1000 Genomes Project [28] will be capable of yielding more accurate estimations on the amount of common copy number variation. This is especially important for Sub-Saharan African populations that show higher degrees of genetic variation [29]. In order to render the calling of single CNVs more accurate and to overcome the limitations of the current genotyping platforms, it is mandatory to identify population-specific CNVRs and to enrich the marker density for these known CNVRs on the respective custom arrays.

In summary, we generated comprehensive CNVR-maps using micro-arrays in two cohorts of ethnically distinct individuals from Switzerland and Sub-Saharan Africa. The maps represent an extendable framework that can leverage the detection of common CNVs and additionally assist in interpreting CNV-based association studies.

## Methods

### Ethics Statement

#### Swiss sample

After complete description of the study to the participants, written informed consent was obtained. The ethics committee of the Canton of Zurich, Switzerland specifically approved this study.

#### Rwandese sample

Because most of the study subjects were analphabetic, they were fully informed verbally before participation. The informed consent was also in verbal form for the same reason. Both the ethical boards of Mbarara University of Science and Technology, Uganda and the University of Konstanz, Germany specifically approved this study.

Research permit for the study was obtained by the Ugandan government:

PRESIDENT'S OFFICE

RESEARCH SECRETARIAT

P.O BOX 7168

KAMPALA, UGANDA

Research Identity Cards were issued to the researchers by the Uganda National Council for Science and Technology, P.O. Box 6884 Kampala granting them access to the Nakivale refugee camp, which is a restricted area that can only be accessed for persons in possession of the according permits.

### Sample collection and SNP Genotyping

We recruited a total number of N = 1167 individuals from two ethnically distinct populations from Switzerland and Rwanda. The Swiss population consisted of 717 healthy, young individuals. The Rwandese sample is comprised of 450 survivors of the Rwandese Civil War recruited in the Nakivale refugee camp, Uganda [30]. DNA was isolated from whole blood or from saliva. Samples were genotyped using the Affymetrix Genome-Wide Human SNP Array 6.0 following manufacturer's recommendations. Consecutive CNV analysis was performed with the Birdseye and the PennCNV algorithm. For downstream statistical analysis only samples surviving all Quality Control (QC) Criteria were used and analysis was generally restricted to autosomal chromosomes (see Methods S1 for a detailed description QCs and the mapping procedure).

Two randomly chosen subsets consisting of 9 individuals of the Swiss population and a subsample comprising 7 Swiss and 6 Rwandese individuals were processed on the Nimblegen 385K aCGH array and the Illumina 1M-Duo BeadChip, respectively. Data analysis of the Nimblegen 385K aCGH array was conducted applying the wuHMM algorithm [12]. Data of the Illumina arrays was processed with Bead Studio (Illumina's proprietary software). CNV analysis was done applying PennCNV.

The screening sample that was used for gross validation of methods consisted of three individuals with previously verified large-scale aberrations. DNA for these three subjects was isolated from whole blood and hybridized to the Affymetrix Genome-Wide Human SNP Array 6.0. Data of the validation sample was analyzed using Birdseye, PennCNV and the CNAM algorithm of the commercial Software package Helix Tree [19].

### Statistical Analysis

Statistical analysis and plots were done using R [31]. Data reported by the applied CNV finding algorithm on length and frequency of CNVs that was used to analyse population differences, were not normally distributed as indicated by Shapiro-Wilk tests. Therefore Mann-Whitney-U tests were used. For comparison of populations, subjects with more than 500 reported CNVs were removed from statistical analysis, since we assumed that in these cases high misclassification or overfragmentation of CNVs is present. Inclusion of these individuals resulted in even lower p-values (data not shown).

## Supporting Information

Figure S1 **This workflow chart is depicting the proceedings of the CNV congruency check and the merge process.** We first applied an overlap-check for the CNVs reported by the different algorithms. If CNV events had overlaps, copy number state (gain vs. loss) was also evaluated. Out of all CNVs detected, only 1916 events in the Swiss sample and 324 CNVs in the Rwandese sample had to be removed due to incongruent direction of effect. This means that in the Swiss sample a total of 858 overlapping CNVs had been reported as loss by one algorithm and as gain in copy number variation by the other algorithm. In the Rwandese sample, this was the case for only 162 events. All CNV events that had not at least partially been detected by the second algorithm were discarded. A total of 22,700 “merged” CNV events survived this quality control in the Swiss sample and 19,969 CNVs in the Rwandese population, respectively.
(EPS)

Methods S1 (DOC)

Table S1 (XLSX)

Table S2 (XLSX)
