# Supplementary material for: Microarray-Based Maps of Copy-Number Variant Regions in European and Sub-Saharan Populations
Source: PLoS One. 2010 Dec 16;5(12):e15246. doi: 10.1371/journal.pone.0015246 (PMC3002949; doi:10.1371/journal.pone.0015246)
Supplement: Methods S1 — (DOC) [file pone.0015246.s002.doc]

**Supplementary Methods**

***Samples***

Swiss sample: We recruited 717 healthy, young Swiss individuals. DNA was isolated from whole blood or from saliva. Blood was drawn by using 2 x 9ml EDTA tubes (Sarstedt, Germany), DNA was extracted using the QIAamp DNA blood maxi kit (Qiagen, Hilden, Germany). For DNA extraction form saliva, the Oragene® DNA sample collection kit was used.

Rwanda sample: 478 survivors of the Rwandan civil war were recruited in the Nakivale refugee camp, Uganda. For DNA extraction form saliva, the Oragene® DNA sample collection kit was used.

***Array-based genotyping***

Samples were processed as described in the Genome-Wide Human SNP Nsp/Sty 6.0 User Guide (Affymetrix). Briefly, genomic DNA concentration was determined by using a Nano-Drop ND-1000 and adjusted to 50ng/ul in water. 250ng of DNA was digested in parallel with 10 units of Sty I and Nsp I restriction enzymes (New England Biolabs, Beverly, MA) for 2 hours at 37°C. Enzyme specific adaptor oligonucleotides were then ligated onto the digested ends with T4 DNA Ligase for 3 hours at 16°C. After adjustment to 100ul with water, 10ul of the diluted ligation reactions were subjected to PCR. Three PCR reactions of 100ul were performed for Sty digested products and four PCR reactions for Nsp. PCR was performed with Titanium Taq DNA Polymerase (Clontech, Mountain View, CA) in the presence of 4.5 μM PCR primer 002 (Affymetrix), 350 μM each dNTP (Clontech), 1M G-C Melt (Clontech), and 1X Titanium Taq PCR Buffer (Clontech). Cycling parameters were as follows, initial denaturation at 94°C for 3 minutes, amplification at 94°C for 30 seconds, 60°C for 45 seconds and extension at 68°C for 15 seconds repeated a total of 30 times, final extension at 68°C for 7 minutes. Reactions were then verified to migrate at an average size between 200-1100 bps using 2% TBE gel electrophoresis. PCR products were combined and purified with the Filter Bottom Plate (Seahorse Bioscience, North Billerica, MA) using Agencourt Magnetic Beads (Beckman Coulter, Fullerton, CA). Purified PCR products were quantified on a Zenith 200rt microplate reader (Anthos-Labtec, Cambridge, UK). 4 to 5 ug/ul were obtained on average for each sample. From this stage on, the SNP Nsp/Sty 5.0/6.0 Assay Kit (Affymetrix) was used. Around 250 ug of purified PCR products were fragmented using 0.5 units of DNAse I at 37°C for 35 minutes. Fragmentation of the products to an average size less than 180 bps was verified using 4% TBE gel electrophoresis. Following fragmentation, the DNA was end labeled with 105 units of terminal deoxynucleotidyl transferase at 37°C for 4 hours. The labeled DNA was then hybridized onto Genome-Wide Human SNP 6.0 Array at 50°C for 18 hours at 60 rpm. The hybridized array was washed, stained, and scanned according to the manufacturer’s (Affymetrix) instructions using Affymetrix GeneChip Command Console (AGCC, version 0.0.0.676).

***Quality Control***

All samples underwent stringent quality control. After processing of all samples with the Affymetrix Genome Wide Human SNP 6.0 we excluded all samples from further analysis with contrast-qc < 0.4 and qc-call-rate < 0.86 according to manufacturers recommendations. All samples passing these QC-criteria were analyzed with the Birdsuite Software package provided on the BROAD Institute website. (<http://www.broadinstitute.org/science/programs/medical-and-population-genetics/birdsuite/birdsuite-0>)

We then used average estimates of copy number genome wide (genome-wide =chr1-22), which is reported by Birdsuite and removed any sample with genome-wide “overall_cn_estimates” values greater or less than three standard deviations from the average estimate of copy number. Further we eliminated any sample with a high sample-specific measure of noise in the signal intensity of SNP and CN probes (> 3 standard deviations). This data is provided in the snp_sample_variances.txt and cn_sample_variances.txt output files generated by the Birdsuite. Additionally we excluded CNVs that comprised less than one marker per 10kb, since this QC eliminates CNVs that erroneously span the whole centromeric region of chromosomes.

For statistical analysis only samples surviving all QC Criteria were used and were generally restricted to autosomal chromosomes.

***Application of CNV algorithms***

The Birdseye algorithm was run as part of the Birdsuite. Unfortunately Birdseye lacks the opportunity to change the default settings, which could be useful in cases where there is evidence for fragmentation of CNVs (*Figure 2)*. We would encourage the authors of Birdseye to add an additional feature, which allows manual setting of parameters. PennCNV was applied with default settings using the built in correction for GC-content related waviness.

***Construction of the CNVR-Map***

We checked for overlaps between the results yielded by Birdseye and PennCNV using “compare_cnv.pl” (a Perl program supplied with the PennCNV-package). The program outputs all overlapping regions algorithm-wise. We then checked for congruent direction of effect in copy number state per individual (gain-loss coded) and removed all incongruent copy number events. Congruent CNVs were used to construct CNVR maps per population. All congruent CNVs were merged into one CN-event using the outer boundaries of the two CNVs. CNVRs are created using all regionally overlapping CNVs of all individuals and identifying the outer boundaries, which include all CNVs found in that genomic area. All single CN-events were removed, so that CNVRs consist of at least two overlapping CN-events that were found by both algorithms in at least two individuals. After processing the Swiss and the Rwandese population separately, we ran the CNVR finding procedure using the Rwandese and Swiss CN-events, that were detected by both algorithms and checked for congruency beforehand. This way we built a CNVR map that contains genomic regions prone to structural variation in two populations with differences in genetic diversity.

Congruency check and generation of CNVRs was based on customized Perl-scripts available for download on http://palenque.lcg.unam.mx/~hsamano/proyecto/cnv_drift_index.php.
